# Nisin and rutin as potential coating agents for iron oxide nanoparticles for enhanced theranostic applications against cancer

**DOI:** 10.1038/s41598-026-49686-7

**Published:** 2026-05-02

**Authors:** Omnia A. Saad, Abdo A. Elfiky, Mohamed M Fathy, Ibrahim M. Ibrahim, Mohamed A. Ibrahim, Ahmed M. Elgharib, Omnia A. Ali, Ahmed A. Ezat

**Affiliations:** Department of Biophysics, Faculty of Sciences, Cairo University, Giza, 12613 Egypt

**Keywords:** GRP78, IONP, characterization, IONP-protein interaction, membrane permeability, rutin, nisin, surface coating, breast cancer, Biochemistry, Biotechnology, Cancer, Chemistry, Drug discovery, Nanoscience and technology

## Abstract

Iron oxide nanoparticles (IONPs) have proven to be of therapeutic potential against cancer. The feature of the surface coating can affect important properties of IONPs; it is therefore critical for further understanding how these materials react to physiological conditions, which is still needed to fully exploit the potential of IONPs for their theranostic applications. In this study, we explored the therapeutic potential of rutin and nisin conjugated IONPs as anticancer agents. One important hallmark of many cancers is the overexpression of the endoplasmic reticulum-resident chaperone, GRP78, and its translocation to many cellular compartments, including the cell membrane. We explored the potential binding affinity of rutin and nisin against the substrate-binding domain β (SBDβ) of GRP78. The results show promising results for both nisin and rutin, with more enhanced binding capability of the former due to its extended structure (peptide in nature), forming more non-bonded interactions with the GRP78 surface. Our findings pave the way for the use of these coating agents against the cell-exposed chaperone, GRP78, to alleviate its chemoresistance characteristics in cancer.

## Introduction

Surface coating of NPs can be an essential way to enhance their potent therapeutic effect for various medical applications. Using natural coating agents on the NP’s surface will preserve their physicochemical stability^1^. By modifying the NPs’ surface with particular ligands, NPs can target particular cells or tissues inside the body. This targeted approach maximises therapeutic benefits and minimises side effects by enabling more accurate drug delivery^2^.

In many medical applications, including magnetic resonance imaging, medication and gene delivery, cancer therapy, and catalysis, iron oxide nanoparticles (IONPs) are essential^3^. Since they have been permitted by the US Food and Drug Administration (FDA) and are relatively simple to formulate, IONPs are regarded as the most promising nano-formulation among the several forms of magnetic nanoparticles. The FDA has approved IONPs for several uses, including treating iron-deficient anaemia and as contrast agents for imaging liver lesions and lymph node metastases^3^. The use of a natural stabilizing coating for IONPs is recommended to improve their biocompatibility, facilitate more specific targeting, and prolong their circulation time by lowering their clearance rate via the reticuloendothelial system^3^.

Rutin (Ru) is an organic capping agent that can alter the surface of IONPs. It is considered a naturally occurring citrus flavonoid with a polyphenolic structure (4). Ru demonstrates significant anti-viral efficacy against a range of viral pathogens, positioning it as a viable phytotherapy for COVID-19. Among the different flavonoids tested for their effectiveness against COVID-19, Ru showed the highest binding affinity to the protease of SARS-CoV-2, indicating it as the most advantageous drug target for the treatment of COVID-19^5^.

Nisin, a cationic peptide consisting of 34 amino acid residues, is naturally produced by *Lactococcus lactis* subsp. Lactis. It exhibits antimicrobial activity against Gram-positive bacteria and non-toxicity towards humans^6,7^. Researchers have demonstrated the antibacterial capabilities of iron oxide magnetic nanoparticles coated with nisin and nystatin against Gram-positive bacteria^7,8^. This implies that these functionalised nanoparticles could be used as antimicrobials in cutting-edge, novel technologies involving magnetic nanomaterials^8^.

GRP78, also known as immunoglobulin heavy chain binding protein (BiP), which is a member of the Heat Shock Protein 70 (HSP70) family of chaperone proteins (HSPA5), can be found on the Endoplasmic Reticulum (ER) of eukaryotes. GRP78, consisting of 654 amino acids, is known for its function as a molecular chaperone in the ER^9,10^. As a chaperone, GRP78 corrects protein folding and prevents the transport of incorrectly folded proteins by directing the misfolded proteins for refolding to keep the unfolded protein concentration at a minimum or move it to the degradation mechanisms^11^. GRP78 is one of the host-cell receptors for the SARS-CoV-2 spike protein. Experimentally, it has been shown that it helps in the entry of the virus into the host cell. The residue T453 in GRP78 is a key for interaction with the spike protein, as presented experimentally by mutating it into Aspartic acid (T453D)^12^. T453 is found in the substrate-binding domain of GRP78.

## Materials and methods

### Chemicals

Iron (III) chloride hexahydrate (FeCl_3_·6H_2_O), ammonium hydroxide (NH_4_OH), ferrous sulfate heptahydrate (FeSO_4_.7H_2_O), nisin, rutin, sodium citrate dihydrate, and ethanol were all acquired from Sigma-Aldrich, USA. Gibco (USA) was the supplier of dimethyl sulfoxide (DMSO).

### Methods

#### Preparation of rutin-capped iron oxide nanoparticles (R-IONP)

Fe (III) and Fe (II) salts with a molar ratio of 2:1 were dissolved in 40 ml of water with vigorous mechanical stirring. At 40 °C, 1 ml of Rutin solution (30 mg Rutin dissolved in 1 ml DMSO) was added dropwise. At 80 °C, 5 ml of 28% ammonium hydroxide was added, and the mixture was magnetically stirred for 30 min until a distinct brown shade appeared (R-IONP). A permanent magnet was used to isolate Rutin-IONP and was washed many times with deionized water and ethanol to eliminate unreacted contaminants. After 4 h of drying in the oven at 60 °C, INPs were finally obtained. After separation of R-IONP with a permanent magnet, the concentration of the collected free Rutin in the supernatant was calculated at an excitation wavelength of 380 nm and emission wavelength of 490 nm using a spectrofluorometer (Shimadzu, RF5301pc, Japan). Then, the coating efficiency was calculated using the following formula:

$$Coating{\text{ }}efficiency{\text{ }}\left( \% \right)=\frac{{Initial{\kern 1pt} \, amount\,\,of\,\,Ru\, - \,Supernatent\,free\,\,amount\,\,of\,\,Ru}}{{Initial\,\,amount\,\,of\,\,Ru}}$$

#### Preparation of nisin-capped iron oxide nanoparticles (N-IONP)

N-IONP might be made via post-functionalization synthesis of citrate IONP capped with nisin. Fe (III) and Fe (II) salts with a molar ratio of 2:1 were dissolved in 40 ml of water with vigorous mechanical stirring. At 80 °C, 5 ml of 28% ammonium hydroxide was added dropwise, then 4.9 g of sodium citrate was added. This was accomplished by combining 30 µl of nisin with the IONP. A permanent magnet was used to isolate N-IONP, and it was washed many times with deionized water and ethanol to eliminate unreacted contaminants. After 4 h of drying in the oven at 60 °C, INPs were finally obtained. The coating efficiency of nisin was calculated with the same previous method at an excitation wavelength of 280 nm, and observing emission at approximately 350 nm.

### Physical characterization

#### Transmission electron microscope (TEM)

A transmission electron microscope (TEM) (JEM 1230 electron microscope, Jeol, Tokyo, Japan) was used to determine the size and morphology of the produced nanocomposites. TEM is a technique that involves passing an electron beam through an extremely thin object. It operates on the same principles as a light microscope, except that rather than light, it uses electrons. A drop of solution was applied to a copper-coated carbon grid. Before the examination, the grid had to be dried at room temperature for 5 min.

#### Atomic force microscope (AFM)

Topographic information was acquired with the atomic force microscope (AFM) (Wet—SPM9600, Japan). A tip scans a sample for AFM, and while scanning, the deflection of the cantilever on which the tip is positioned determines the force between the tip and the sample. Drawing the deflection of the cantilever against its position on the sample produces a topographic image.

#### Dynamic light scattering (DLS)

DLS measures the hydrodynamic size of particles and molecules in suspension. The measured hydrodynamic diameter reflects the dimension of the NP along with a layer of surface-bound solvent. The size measured by DLS was determined to be the “hydrodynamic diameter,” which is the size of a hypothetical hard sphere that diffuses in the same manner as the particle being measured. The Zeta Sizer (NICOMP TM 380 ZLS, USA) was used to measure the size distribution of the generated nanoformulations.

#### Zeta potential

Zeta potential was assessed utilizing the zeta sizer nano series (Nano ZS, Malvern Instruments, Malvern, UK) within a potential range of -200 to 200 mV. The potential evaluation was conducted at 25 °C, and the zeta potential value was derived by analyzing the direction and velocity of the prepared nanoformulations in the applied electric field.

#### Fourier transform infrared (FTIR) spectroscopy

FTIR spectroscopy was used to determine the functional groups of the active ingredients in the produced nanoformulations by examining the peak value in the infrared radiation area. An FTIR spectroscope (FTIR Edwards High Vacuum, Craeley Sussex, England) was used to get the transmitted peaks in the range of 449.33 to 4000.6 cm^− 1^.

#### Cell culture

MDA-MB-231: Breast cancer was obtained from Nawah Scientific Inc. (Mokatam, Cairo, Egypt). Cells were maintained in DMEM media supplemented with 100 mg/mL of streptomycin, 100 units/mL of penicillin, and 10% of heat-inactivated fetal bovine serum in a humidifier, 5% (v/v) CO_2_ atmosphere at 37 °C.

#### Cytotoxicity assay

Cell viability was assessed by the sulforhodamine B (SRB) assay. Aliquots of 100 µL cell suspension (5 × 10^3^ cells) were placed in 96-well plates and incubated in complete media for 24 h. Cells were treated with another aliquot of 100 µL media containing drugs at various concentrations. After 48 h of drug exposure, cells were fixed by replacing the media with 150 µL of 10% TCA and incubated at 4 °C for 1 h. The TCA solution was removed, and the cells were washed 5 times with distilled water. Aliquots of 70 µL SRB solution (0.4% w/v) were added and incubated in a dark place at room temperature for 10 min. Plates were washed 3 times with 1% acetic acid and allowed to air-dry overnight. Then, 150 µL of TRIS (10 mM) was added to dissolve protein-bound SRB stain; the absorbance was measured at 540 nm using a BMG LABTECH^®^- FLUOstar Omega microplate reader (Ortenberg, Germany).

### Statistical analysis

By triplet repetition of each experiment, results were collected as average ± SD. Using SPSS, one-way analysis of variance (ANOVA) was performed for the comet test and oxidative stress parameters. Scheffe’s test was used to compare the independent and dependent parameters at statistically significant values (*P* < 0.05) as a post-hoc comparison.

### In silico analysis

#### GRP78 structural preparation

The structure of GRP78 was retrieved from the protein data bank using PDB ID 5E84^13^. Water molecules and ions were removed except for the two Zn ions and the ATP molecule in the N-terminal. Only one chain was used from the downloaded structure. To add the glycans to the protein at T85, T151, T166, T184, and T203^14^, the CHARMM-GUI website was utilized^14–18^.

#### MD simulation

After adding the glycans to the protein, the CHARMM-GUI webserver was used to prepare the systems and generate the input files for the GROMACS MD engine^19,20^. A standard unbiased MD simulation lasting 500 ns was performed in GROMACS 2021 to sample the conformation of the protein. We utilized the solution builder module of the CHARMM-GUI server to generate the input files^21–24^. The GRP78 protein was solvated in a 13.2 nm long cubic box. After solvating the system using the transferable intermolecular potential 3 points (TIP3P) water model with a padding of 1 nm from the furthest distant atom, the systems were neutralized by adding NaCl ions at a concentration of 0.154 M. The CHARMM36m force field was used to obtain the amino acid parameters of the protein, as well as those of the TIP3P water model and the ions. The ATP molecule was parameterized using the CHARMM general force field (CGenFF).

Periodic boundary conditions (PBC) were used in all three dimensions throughout the simulation. To prevent atomic collisions, the potential energy was minimized using the steepest descent algorithm. Following that, the temperature and pressure in the systems were equilibrated in two steps. When the maximum force applied to any atom was less than 100 KJ/(mol.nm), or when the number of reduction steps reached 100,000, the minimizing process was regarded to be converged. The NVT ensemble and the velocity rescale technique were utilized to reach an average temperature of 310 K during the first phase of the equilibration process. The NPT ensemble, the Berendsen barostat, and the velocity rescale algorithms were utilized in the second phase to maintain an atmospheric pressure of 1 atm and an average temperature of 310 K^25^. For the 500 ns production run, an NPT ensemble was utilized, and the temperature and pressure were regulated by a Nose-Hoover thermostat and a Parrinello-Rahman barostat, respectively. The temperature was kept at 310 degrees Celsius, while the pressure was maintained at one atmospheric pressure^26^. The LINear Constraint Solver (LINCS) was used to impose length constraints on the hydrogen-bonded atoms^27^. We utilized Particle Mesh Ewald (PME) summation to calculate the electrostatics with a threshold of 1.2 nm^28^. By using a time step of 1 femtosecond during equilibration and 2 femtoseconds throughout the production run, the Newtonian equations of motion were integrated using the leap-frog approach. We took 5,000 frames at 0.1 ns intervals during the simulation.

#### Clustering and docking of nisin, rutin, and citrate to GRP78

The simulation of GRP78 was clustered based on the RMSD of Cα atoms using the TTClust Python library to obtain a representative frame for each cluster^22^. The number of clusters was determined based on the elbow method, which generated two clusters. The representative frame for each cluster was selected as the middle frame. Two representative frames were obtained for GRP78. For each frame, it was loaded in Autodock Tools V 1.5.7 and was prepared by removing nonpolar H-atoms and adding Gasteiger charges. For GRP78, the searching box was defined to encompass the SBDβ domain with dimensions and center of x = 70, y = 120, z = 120, and x = 20.464, y = 53.937, z = 53.097, respectively, with a spacing of 0.686. We include enough space for small molecules and nisin conformational sampling^30,31^. The exhaustiveness in AutoDock Vina was set at 1000 for an elaborate search of the best pose^30,31^. Rutin, citrate, and nisin (PDB ID: 1WCO) were prepared using the same program and were prepared using the same steps. However, for nisin, only ten dihedrals (Ile1 Φ and ψ, Dhb2 Φ and ψ, D-Ala3 Φ, Cys7 ψ, D-Abu8 Φ, Cys11 ψ, Lys12 Φ and ψ) were allowed to rotate while the rest were kept non-rotatable^32^. The results from docking were examined by eye, and the best pose in each of the six systems was used as a starting point to perform the MD simulation for 200 ns using GROMACS by utilizing the same options as mentioned above. The CHARMM36 force field parameters of nisin were obtained from a previous work^33^.

#### MM-GBSA

The binding free energy (ΔG) between each ligand and the GRP78 protein was computed using the Molecular Mechanics/Generalized Born Surface Area (MM-GBSA) method, as implemented in the gromacs molecular mechanics Poisson Boltzmann surface area (gmx_MMPBSA) software package version 1.6.4 (https://valdes-tresanco-ms.github.io/gmx_MMPBSA/dev/)^34,35^. This approach integrates molecular mechanics energies with continuum solvation models to estimate the free energy of binding. To elucidate the contributions of individual amino acid residues to the overall binding affinity, per-residue energy decomposition analysis was carried out for all residues located within a 1 nm cutoff from the ligand.

All calculations were performed under physiological ionic strength conditions (0.154 M NaCl), employing a Generalized Born (GB) solvation model with the GB parameter (*igb*) set to 5. The internal and external dielectric constants were assigned values of 1.0 and 78.5, respectively, to reflect the polarizability differences between the solute and the surrounding aqueous environment. The fundamental formulation used for the binding free energy calculation is described by Eq. 1:

1 $$\Delta{{G\; = \; < \;Gcomplex\; - \;(Greceptor\; + \;Gligand) > \;\;\;\;}}$$

Here, < > denotes the ensemble average of the free energies for the complex, receptor, and ligand calculated across the analyzed simulation frames. In this study, the entire 200 ns trajectory was utilized, with snapshots sampled every 10 frames, resulting in a total of 200 frames for the energy calculations. The various energy components can be determined according to Eq. 2 through 6:

2 $$\Delta{{Gbinding\; = \;}}{{{\Delta}H\; - \;T}}\Delta{{S\;\;}}$$

3 $$\Delta{{H\; = \;}}{{E_{gas}}}{{\; + \;}}{{E_{sol}\;\;}}$$

4 $$\Delta{{E_{gas}\; = \;}}\Delta{{E_{ele}}}{{\; + \;}}\Delta{{E_{vdw}\;\;}}$$

5 $$\Delta{{E_{sol}\; = \;}}\Delta{{E_{GB}}}{{\; + \;}}\Delta{{E_{SA}\;\;}}$$

6 $${{ESA\; = \;\gamma }}\cdot{{SASA\;\;\;\;}}$$

where: ΔH represents the enthalpy, which is derived from the gas-phase energy (E_gas_​) and the solvation-free energy (E_sol_​). The term −TΔS accounts for the entropic contribution to the binding free energy, and it was not calculated in this study. The gas-phase energy (E_gas_​) comprises electrostatic (E_ele_​) and van der Waals (E_vdw_​) energy terms. The solvation-free energy (E_sol_​) is calculated as the sum of the polar solvation energy (E_GB_​) and the nonpolar solvation energy (E_SA_​), with the latter estimated based on the solvent-accessible surface area^36,37^.

## Results and discussion

**Fig. 1:**
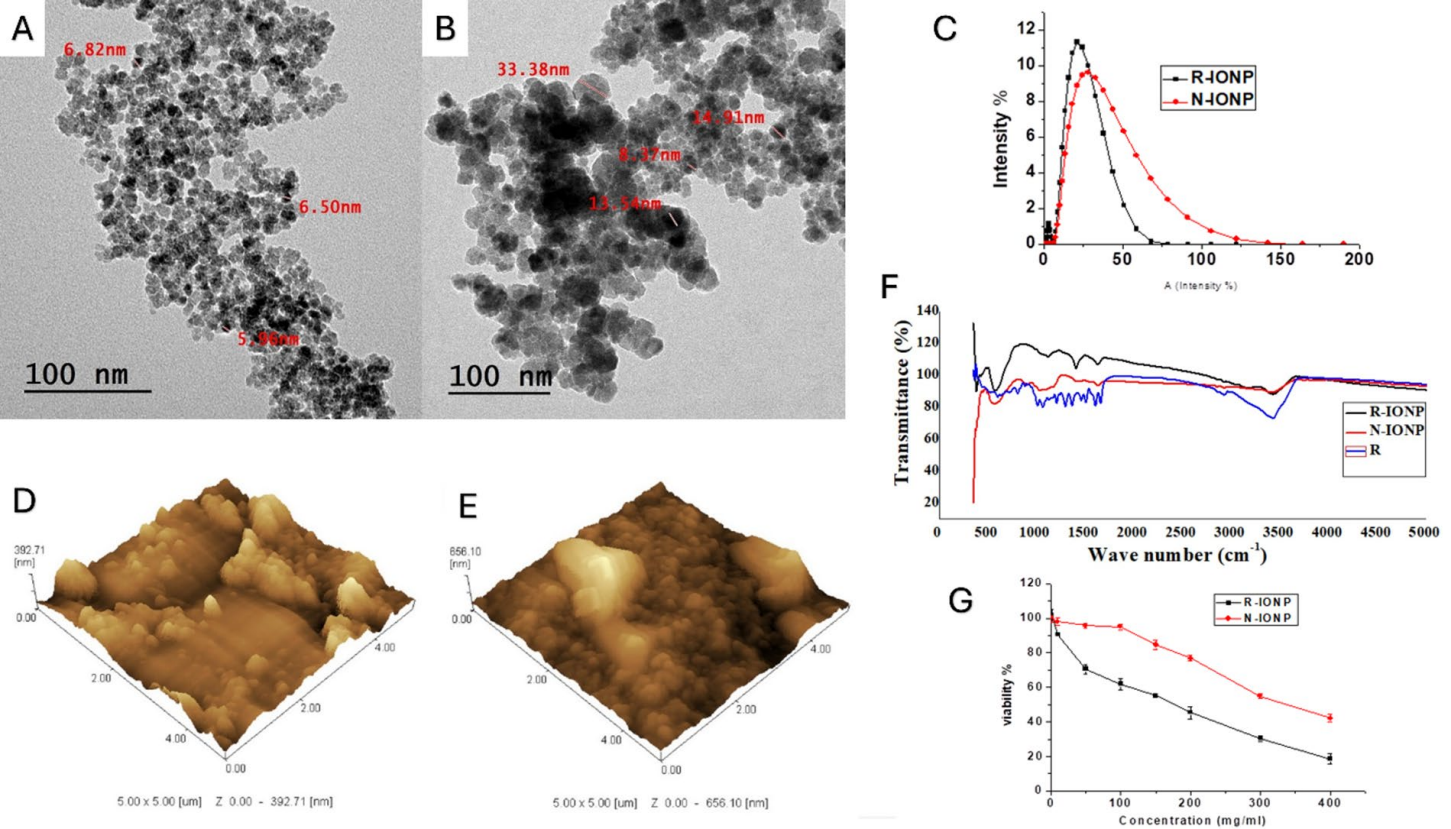
Characterization analysis of the formulated IONPs. (**A**) and (**B**) Transmission electron microscopy micrographs of R-IONP and N-IONP, respectively. The scale bars represent 100 nm. (**C**) The dynamic light scattering graph shows the hydrodynamic size distribution of both N-IONP and R-IONP. (**D**) and (**E**) Atomic force microscopy micrographs of R-IONPs and N-IONPs, respectively. (**F**) The Fourier-transform infrared spectroscopy analysis of rutin (blue), R-IONP (black), and N‐IONP (red). (**G**) The cell survival curve represents the effect of different concentrations of R-IONP and N‐IONP on the viability of MDA-MB-231 breast cancer cells, determined by SRB assay.

The spherical shape of R-IONP and polyorphological shape of N-IONP were clarified in Fig. 1. The mean diameter was about 6.4 ± 0.29 nm and 17.54 ± 5.5 nm for R-IONP and N-IONP, respectively. TEM images in Fig. 1A and B represent an aggregation pattern within N-IONP, while there is a homogeneous size within R-IONP. The dynamic light scattering analysis results showed that the average particle sizes of R-IONP and N-IONP were 24.4 ± 3.5 and 29.6 ± 4.6 nm, respectively (Fig. 1C). For R-IONP and N-IONP, it is clear that the samples have a crystalline structure with discontinuous rings in their electron diffraction patterns (Supplementary Fig. S1). The small particle size is what causes the discontinuous rings in the diffraction^38^. The crystalline structure of the IONP was confirmed by XRD analysis (Fig. S2, Supplementary Information), showing characteristic peaks corresponding to magnetite at the values of 30.65 ^°^, 35.56 ^°^, 43.41 °, 57.69 ^°^, and 63.8 ^°^.

Additionally, the topographic information and Surface roughness of prepared nano formulas have been investigated using AFM (Fig. 1D and E). Surface roughness was about 1.4 ± 0.47 and 1.33 ± 0.5 nm for R-IONP and N-IONP, respectively. Surface roughness influences the magnitude of nanoparticle-cell interactions, boosts cellular absorption, facilitates cell adhesion, and promotes cellular uptake^39–41^. Variations in surface roughness may alter the contact potential between the adsorbing molecules and the nanoparticle^42^. The coating effeciancy were 83.56% and 87.75% for R-IONP and N-IONP, respectively.

The polydispersity index (PDI) describes the degree of homogeneity of nanoparticle size distribution. The PDI values were 0.23 and 0.4 for R-IONP and N-IONP, respectively. PDI values indicate a narrow size distribution, while a PDI greater than 0.5 is related to a broad distribution^43^.

On the other hand, zeta potential measurements showed that the net surface charge of R-IONP and N-IONP was negative with an average value of -36.9 ± 4.29, -45.5 ± 4.4 mV, respectively. Zeta potential results indicate that R-IONP is more stable than N-IONP, while a strong zeta potential (either positive (> 30 mV) or negative (<-30 mV)) signifies enhanced electrostatic repulsion among particles, resulting in improved stability and the inhibition of aggregation^44^. Nanoparticles with a net negative surface charge can attach to the cell by binding to cationic plasma membrane sites and then enter the cell via endocytosis^45^. As the surface negativity increased, stability increased^46^, and cellular internalization increased^47^.

The Fourier-transform infrared (FTIR) spectra of R-IONP and N-IONP are represented in Fig. 1F. There is a broad peak around 3400 cm^− 1^, attributed to O-H stretching. This suggests the presence of hydroxyl groups, which may arise from adsorbed water. Furthermore, a peak near 1630 cm^− 1^, likely due to the H-O-H bending of adsorbed water, is consistently detected across all spectra. C = O stretching occurs approximately between 1650 and 1700 cm⁻¹. Aromatic C = C stretching vibrations occur at approximately 1600, 1500, and 1450 cm⁻¹. C-O stretching vibrations occur in the range of 1000–1200 cm⁻¹. The Fe-O stretching band, observed at approximately 570 cm^− 1^, is present in R-IONP and N-IONP spectra, thereby confirming the existence of iron oxide nanoparticles.

### Antitumor activity

The SRB assay was executed on these cells to estimate the antiproliferative actions of the synthesized formulations. The experiment was performed over 48 h of cell incubation. The link between the drug concentration and the cells’ relative viability was leveraged to generate the survival graphs for the cells, and values were shown in Fig. 1G.

SRB assay was executed against MDA-MB-231 cells to estimate the antiproliferative actions of the synthesized formulations (R-IONP and N-IONP). Cells treated with N-IONP at concentrations 10, 50, 100, 150, 200, and 400 µg/ml exhibited cell viability of about 97.94%, 95.87%, 95.08%, 84.6%, 76.86%, 54.98% and 42.06%, respectively. On the other hand, it was interesting that cell viability significantly decreased to 90.62%, 70.58%, 61.99%, 55.17%, 45.34%, 30.45%, and 18.56%, respectively, when they were treated with R-IONP at the same treatment conditions. The IC_50_ of the prepared formulations was 176.9 mg/ml and 358.04 mg/ml R-IONP and N-IONP, respectively.

The results revealed the superior potent therapeutic efficacy of R-IONP than free N-IONP. Both in vivo and in vitro examinations on anticancer mechanisms of Rutin have been widely carried out. It was found that the regulation of different cellular signaling pathways, such as Wnt/β-catenin, p53-independent pathway, PI3K/Akt, JAK/STAT, MAPK, p53, apoptosis, as well as NF-ĸB signaling pathways, helps to mediate the anticancer impacts of this agent^48^. In this study, we explored the potential interaction between rutin and nisin with the GRP78 protein using computational approaches (molecular docking and molecular dynamics simulations), based on the reported role of GRP78 in cancer progression and cellular stress responses^49,50^.

### Dynamics simulations of the tested complexes

**Fig. 2:**
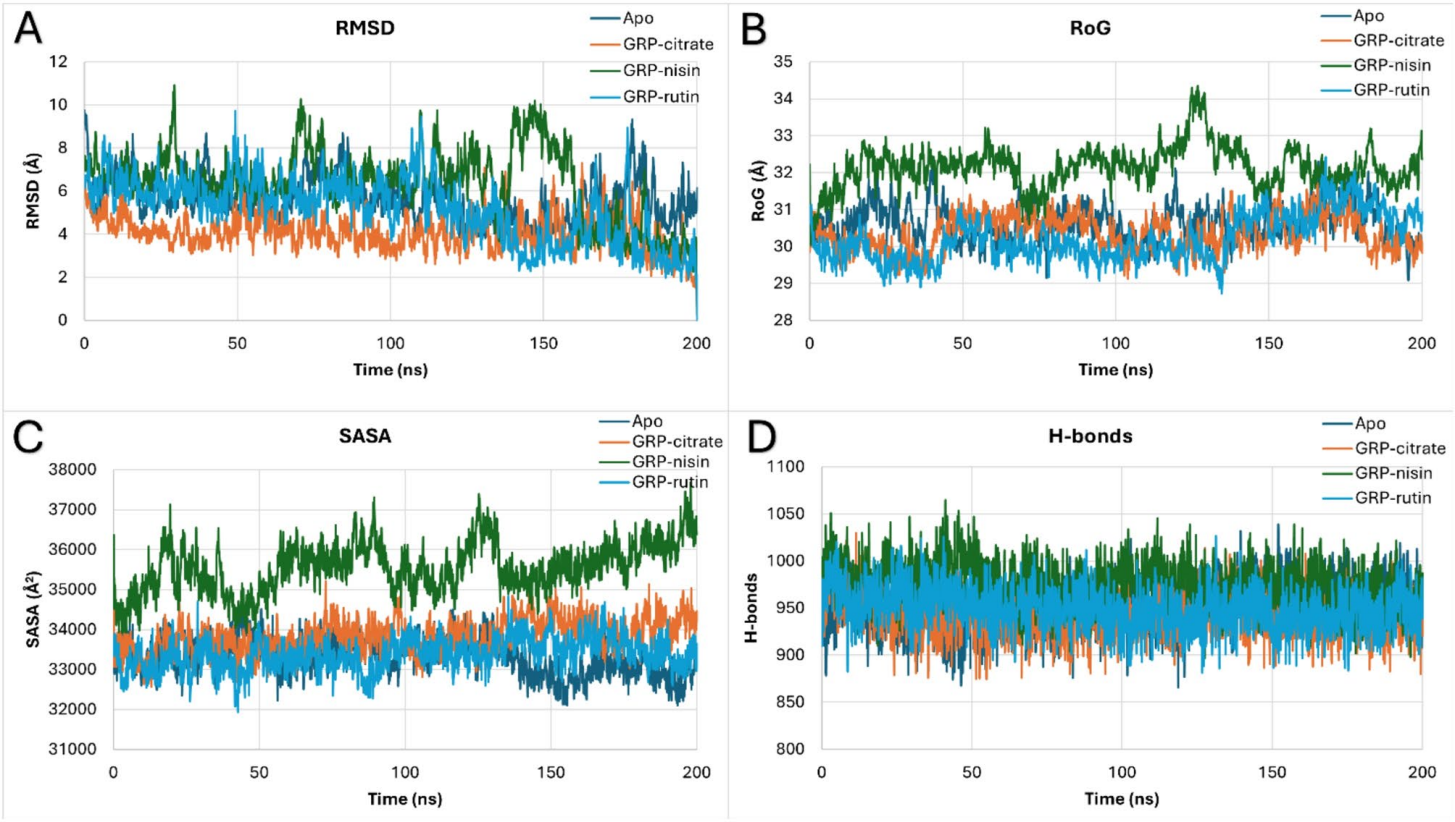

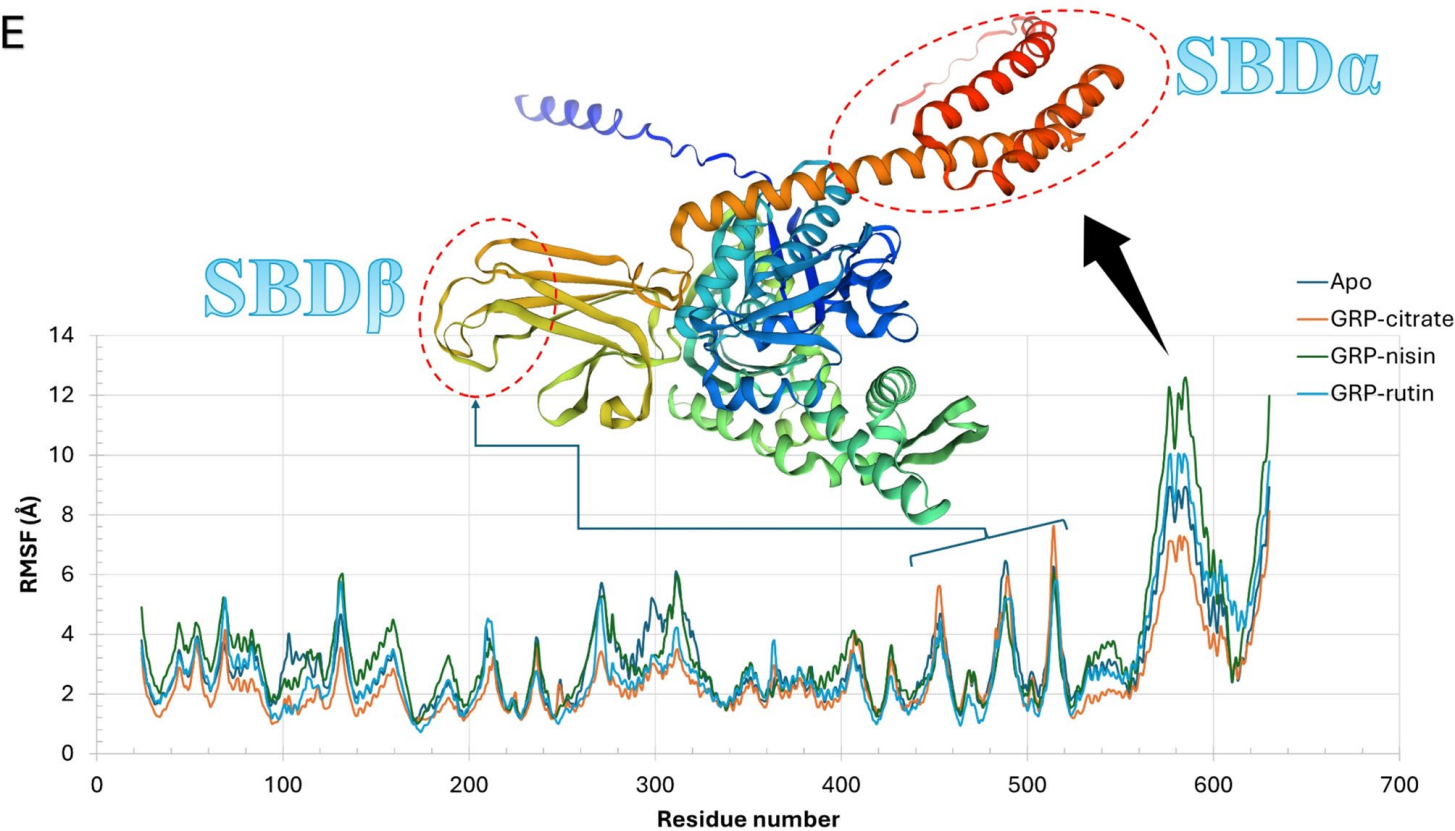
Molecular dynamics simulation analysis during the 200 ns for apo GRP78 (dark blue) and in the complex of citrate (orange), nisin (green), and rutin (blue). (**A**) RMSD in Å, (**B**) RoG in Å, (**C**) SASA in Å^2^, and (**D**) the total number of H-bonds versus the simulation time in ns. (**E**) the residual RMSF in Å alongside the structure of GRP78 in colored carton representation showing the two highly fluctuating regions (SBDα and SBDβ) encircled.

As reflected from the root-mean square deviation (RMSD) curves in Fig. 2A, all the systems are equilibrated during the 200 ns simulation period. Most of the RMSD values lie between 3 Å and 8 Å. The GRP78-nisin system (green) has slightly higher fluctuations compared to other systems. Additionally, the radius of gyration (RoG) curves (Fig. 2B) indicate that the systems are stable during the 200 ns simulation period. Most of the RoG values lie between 29 and 33 Å. Again, the GRP78-nisin system (green) has a higher RoG compared to other systems. This is obvious as nisin (peptide) adds to the system volume compared to the small compounds in the other complexes. Regarding the surface accessible surface area (SASA) curves (Fig. 2C), the systems are stable during the 200 ns simulation period. Most of the SASA values lie between 32,500 and 37,000 Å^2^. Again, the GRP78-nisin system (green) has a higher SASA compared to other systems. This is obvious as nisin (peptide) adds to the system surface area compared to the small compounds in the other complexes. This is also reported in the total number of H-bonds during the simulation period (Fig. 2D). The systems are stable during the 200 ns simulation period, with the H-bond values lying between 900 and 1025.

The per-residue root-mean square fluctuation (RMSF) curves (Fig. 2E) show that the systems are all stable during the 200 ns simulation period. SBDα region of the GRP78 shows the highest fluctuations (6 up to 12 Å), especially for the nisin-GRP78 complex (green), while it is minimum in the case of rutin-GRP78 complex (orange). SBDβ, on the other hand, shows nearly the same fluctuations in all the complexes as the apo protein except for slightly increased values for the rutin-GRP78 complex (orange). These trends of fluctuations reflect the stability of the formed complexes compared to the apo protein during the simulation period.

### The binding energy decomposition (MM-GBSA)

**Fig. 3:**
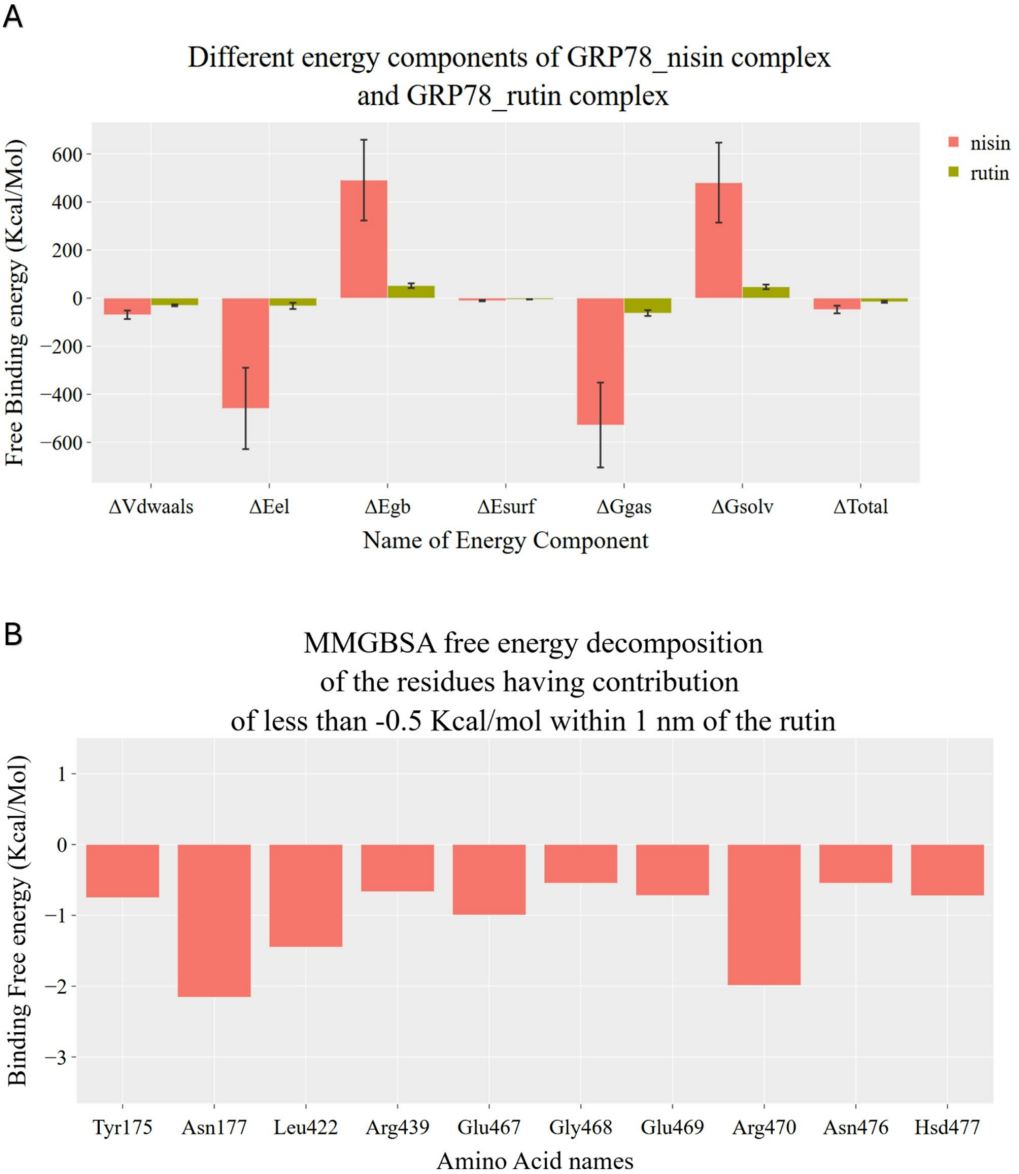

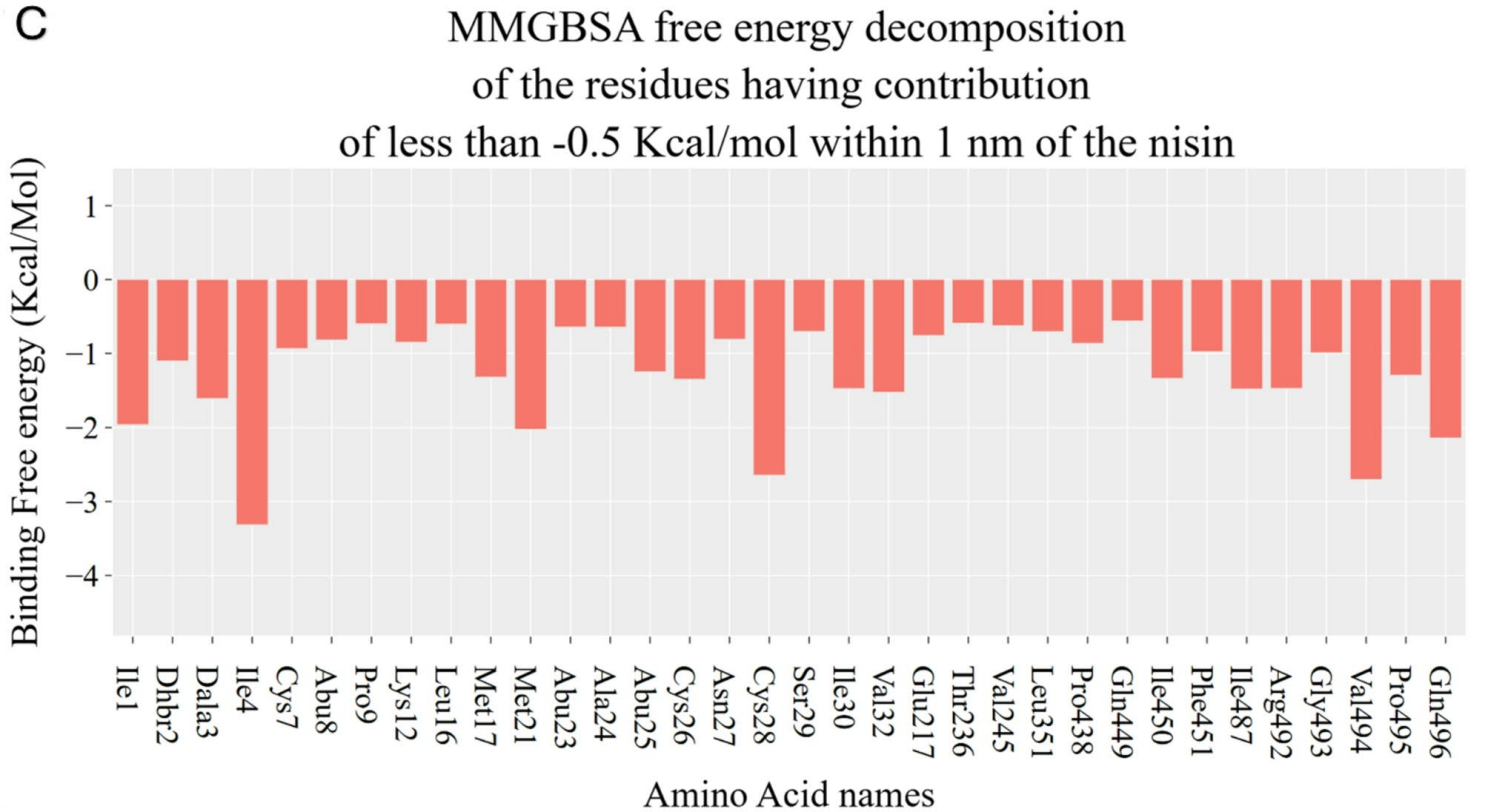
The calculated molecular mechanics-generalized Born surface area (MM-GBSA) for both rutin-GRP78 and nisin-GRP78 systems during the molecular dynamics simulation (MDS). (**A**) shows the energy decomposition for both systems. (**B**) and (**C**) shows the residual energy contribution for rutin-GRP78 and nisin-GRP78, respectively.

The molecular mechanics-generalized Born surface area (MM-GBSA) calculations for rutin-GRP78 and nisin-GRP78 systems are done with the help of the gmx_MMPBSA software package during the entire 200 ns simulations, and results are shown in Fig. 3. The energy decomposition was performed and depicted in the bar graph in Fig. 3A, where the rutin-GRP78 and nisin-GRP78 complexes are shown in green and red bars, respectively. The error bars represent the standard deviations. The total energy differences (ΔTotal) for both nisin and rutin complexes with GRP78 are both negative, which reflects their potential binding partners against GRP78. The ΔTotal of nisin-GRP78 is more negative than the rutin-GRP78 system, which reflects the potential binding capacity of nisin against GRP78. This is mainly due to the nature of the binding partner, as nisin is peptidic in nature have many sites of possible binding potential compared to the small molecule rutin. The residual contribution in binding from GRP78 protein to rutin and nisin is depicted in Fig. 3B and C, respectively. The listed residues contribute with more negative energy values than 0.5 kcal/mol.

For rutin, the most contributed residues from GRP78 that interact with rutin are from the substrate binding domain (SBD), such as L422, E467, and R470, which contribute with values less than 1 kcal/mol in binding. In addition, N177 contributed to binding rutin with a value less than 2 kcal/mol. These residues are either charged (N177, E467, and R470) or hydrophobic (L422), which reflects the formed interaction with rutin.

On the other hand, nisin forms many interactions with GRP78 at both the N-terminal and SBD, as seen in Fig. 3C. The most contributed residues with energy values less than 2 kcal/mol are almost all hydrophobic in nature, such as I1, I4, M21, C28, V494, and E496. These represent the interactions at SBD and the N-terminal domain at the same time due to the extended form of the nisin peptide.

## Data Availability

Data generated during this study are included in the manuscript.
